# Influence of Breastfeeding Factors on Polyamine Content in Human Milk

**DOI:** 10.3390/nu13093016

**Published:** 2021-08-29

**Authors:** Nelly C. Muñoz-Esparza, Edgar M. Vásquez-Garibay, Elizabeth Guzmán-Mercado, Alfredo Larrosa-Haro, Oriol Comas-Basté, M. Luz Latorre-Moratalla, M. Teresa Veciana-Nogués, M. Carmen Vidal-Carou

**Affiliations:** 1 Departament de Nutrició, Ciències de l’Alimentació i Gastronomia, Facultat de Farmàcia i Ciències de l’Alimentació, Campus de l’Alimentació de Torribera, Universitat de Barcelona, Av. Prat de la Riba 171, 08921 Santa Coloma de Gramenet, Spain; nelly.munoz@ub.edu (N.C.M.-E.); oriolcomas@ub.edu (O.C.-B.); mariluzlatorre@ub.edu (M.L.L.-M.); veciana@ub.edu (M.T.V.-N.); 2 Institut de Recerca en Nutrició i Seguretat Alimentària (INSA·UB), Universitat de Barcelona, Av. Prat de la Riba 171, 08921 Santa Coloma de Gramenet, Spain; 3 Xarxa d’Innovació Alimentària (XIA), C/Baldiri Reixac 4, 08028 Barcelona, Spain; 4 Instituto de Nutrición Humana, Universidad de Guadalajara, Salvador Quevedo y Zubieta 750, Guadalajara 44360, Mexico; vasquez.garibay@gmail.com (E.M.V.-G.); eliguzman100@hotmail.com (E.G.-M.); alfredo.larrosa@academicos.udg.mx (A.L.-H.)

**Keywords:** polyamines, putrescine, spermidine, spermine, human milk, full breastfeeding, partial breastfeeding

## Abstract

The polyamine content of human breast milk, which is the first exogenous source of polyamines for the newborn, can be affected by several factors associated with the mother, the infant, or breastfeeding itself. The aim of this study was to evaluate the influence of different breastfeeding factors on the polyamines found in human milk. For this study, a cohort of 83 mothers was considered for up to 4 months, and a subgroup of 33 mothers were followed during the first six months of breastfeeding. Two breast milk samples were collected at each sampling point (foremilk and hindmilk) and the polyamine content was determined by UHPLC-FL. Polyamine levels varied considerably between the mothers and tended to decrease over time. Putrescine was the minor polyamine, whereas spermidine and spermine contents were very similar. The concentrations of the three polyamines were significantly higher in hindmilk than foremilk (*p* < 0.001). Spermidine and spermine levels decreased significantly through the lactation progress (*p* < 0.05). Finally, slightly higher levels of polyamines were observed in the milk of mothers providing partial, rather than full, breastfeeding, although the differences were not significant. The polyamine content in human milk was found to change during a single feed (foremilk versus hindmilk) and as lactation progressed, mainly in response to the specific circumstances of the newborn.

## 1. Introduction

In humans, the intracellular levels of polyamines (i.e., putrescine, spermidine and spermine) are primarily regulated by de novo synthesis, although these bioactive compounds may also have an exogenous origin, mainly from food [1,2]. Breast milk supplies the infant with the first exogenous source of polyamines. Synthesized in the mammary gland during pregnancy and lactation, polyamines are involved in several physiological processes, notably cell growth and differentiation, protein synthesis, RNA transcription, and the regulation of the immune response [3,4,5,6]. Although the required daily intake of polyamines has not been established, their ingestion from human milk is known to be important during the neonatal and infant stages of rapid cell growth [5,7,8].

Human milk polyamines are mostly absorbed in the small intestine by transcellular or paracellular pathways and are subsequently distributed to the different tissues through the systemic circulation [9]. Nevertheless, some of these dietary polyamines remain in the intestines, where they participate in intestinal maturation and improve the integrity of the intestinal barrier [9,10]. Several studies in animal models have shown that polyamines administered orally during the postnatal period induce early maturation of the intestine and act in the repair of the intestinal mucosa [3,10,11,12]. In particular, spermine and spermidine produce morphological changes in the intestinal epithelium and improve mucosal permeability by enhancing the protein expression and enzymatic activity of disaccharidases and alkaline phosphatases [12,13]. At the same time, polyamines play a key role in the development of the immune system. It has been reported that they promote the maturation of intestinal immune cells, increasing the levels of immunoglobulin A in the villi and crypts of enterocytes [10,13,14,15]. The enhancement of intestinal maturation in newborns is consequently associated with a lower intestinal permeability to antigenic macromolecules, thus reducing the risk of developing food allergies [13,16]. Accordingly, Peulen et al. found that a higher spermine intake during the first month of breastfeeding was significantly associated with a reduced incidence of food allergies in children at the age of five [16]. On the other hand, it has also been described that the metabolism of polyamines is involved in adipogenesis [9,17]. In this sense, some authors have shown that the exogenous administration of spermidine or spermine in mice with induced obesity decreased body weight, fat mass, and visceral fat, and stimulated thermogenesis [18,19,20].

**Figure 1:**
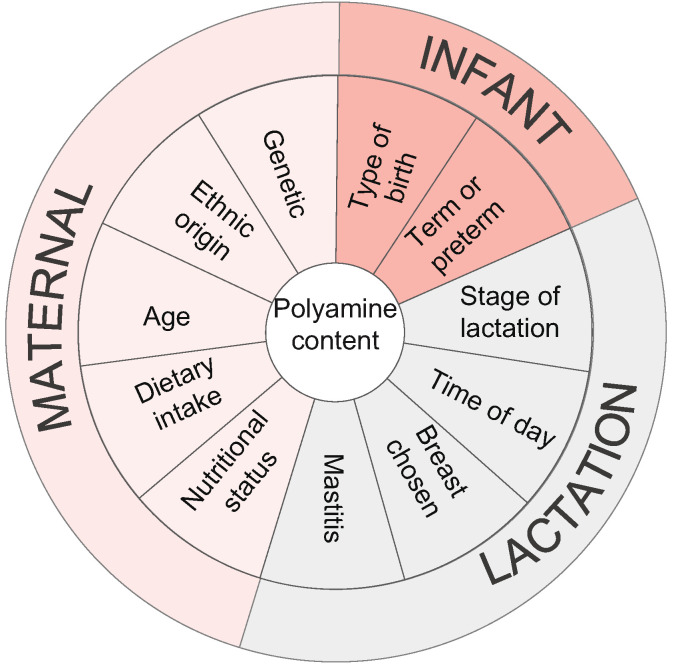
Factors influencing the polyamine content of human milk [6,23,26,27].

Despite the potential health benefits of polyamines in human milk, to date only a few studies have addressed this topic, and they have been based on a limited sample size [3,5,6,21,22,23,24,25]. The highly variable levels of polyamines in human milk reported in the literature could be explained by sample heterogeneity (i.e., they were taken at different phases of lactation) [3,21,22]. Moreover, it has been suggested that the presence of polyamines in breast milk may be influenced by factors intrinsic to the mother, infant, or the act of breastfeeding itself (Figure 1) [5,6]. However, experimental data supporting these hypotheses are still very scarce or nonexistent. Thus, only two studies, carried out by Romain et al. and Pollack et al. in 1992, describe a progressive decrease of polyamines over the course of lactation [21,22], and more recently two studies have found higher polyamine contents in the milk of mothers of pre-term infants compared to the mothers of full-term infants [5,23]. In addition, the influence of maternal diet on the levels of polyamines in human milk has also been addressed by Atiya-Ali et al. [5]; they found that a higher intake of polyamines was associated with increased contents of these compounds in breast milk. 

The aim of this study was to evaluate the influence of different breastfeeding factors on the content of polyamines in human milk. Specifically, the evolution of putrescine, spermidine, and spermine in human breast milk was studied during the first six months of breastfeeding, and levels in foremilk and hindmilk were compared. The effect of the type of lactation (full or partial breastfeeding) and other factors related with the mother–child dyad (age and body mass index of the mother, type of birth, and infant’s birth weight) were also assessed. 

## 2. Materials and Methods

### 2.1. Study Design and Subjects

A non-randomized cohort study was carried out with mothers living in the metropolitan area of Guadalajara, who gave birth at the Nuevo Hospital Civil de Guadalajara “Dr. Juan I. Menchaca” (Mexico) [28]. For this study, a cohort of 83 mothers aged between 18 and 34 years were followed for 4 months, all with a healthy full-term baby with an adequate weight for the gestational age. From this group, a subgroup of 33 mothers were followed during the first six months of breastfeeding. The inclusion and exclusion criteria and the fieldwork strategy are described in detail by Vasquez-Garibay et al. [28]. Briefly, the exclusion criteria were: mothers with a history of chronic, genetic, or congenital diseases; addiction to alcohol, tobacco, or drugs; newborns with congenital malformations and/or genetic diseases.

Full breastfeeding was promoted during the enrollment phase. Overall, 60 mothers provided full breastfeeding (optionally including oral supplements of hydration and/or vitamins/inorganic nutrients) and 23 partial breastfeeding (i.e., the infants were fed with different proportions of breast milk and infant formula).

### 2.2. Human Milk Sample Collection

Breast milk samples were collected using a breast pump that was previously sterilized. At each sampling point, two milk samples of approximately 5–10 mL were collected. The first sample was obtained before the infant was breastfed, corresponding to foremilk (i.e., milk available at the beginning of the feed). The second sample was collected immediately after the infant had been breastfed for 10 min, corresponding to hindmilk (i.e., milk at the end of the feed). All samples were obtained from 9 am to 1 pm, and after collection they were homogenized and stored at −80°C until their analysis.

### 2.3. Analysis of Polyamines in Human Milk

The analysis of polyamines in human milk samples was performed at the Food and Nutrition Campus of the University of Barcelona (Santa Coloma de Gramenet, Spain). Putrescine, spermidine, and spermine were determined by ion-pair ultra-high-performance liquid chromatography coupled to fluorometric detection (UHPLC-FL) as described by Latorre-Moratalla et al. [29]. In brief, 1 mL of homogenized human milk was acidified with 70% perchloric acid and mixed for 20 min. Subsequently, samples were centrifuged (15,000 rpm, 4 °C, 15 min) and the supernatant was recovered and filtered through a 0.22 µm GHP filter (Waters Corp., Milford, MA, USA). Samples were stored at 4 °C until their analysis. Chromatographic separation of polyamines was accomplished using an Acquity UPLC BEH C18 1.7 µm reverse-phase column (2.1 mm × 50 mm) (Waters Corp., Milford, MA, USA), followed by online post-column derivatization with ortho-ophthaldehyde and fluorometric detection (ex: 310 nm and em: 445 nm). The quantification of polyamines in the human milk samples was carried out using the external standard method.

### 2.4. Statistical Analysis

The statistical analysis of data was performed with the IBM SPSS Statistics 25.0 statistical software package (IBM Corporation, Armonk, NY, USA). The data did not follow a normal distribution when analyzed by Kolmogorov–Smirnov and Shapiro–Wilk tests. Thus, the non-parametric Wilcoxon test for paired samples was used to compare the polyamine contents between foremilk and hindmilk and along the lactation process. The comparison between breastfeeding groups was performed with the Mann–Whitney U test for independent samples. In addition, multiple linear regression analysis was performed to evaluate the associations between the polyamine contents in human milk and the characteristics of the mother–child dyad (age and body mass index of the mother, type of birth, weight at birth, and sex of the infant). Values of *p* < 0.05 were accepted as significant.

## 3. Results

Table 1 shows the sociodemographic characteristics of the mother–child dyad, both for the total cohort followed for the first 4 months of lactation (*n* = 83) and for the subgroup of mothers that were followed during the first six months of lactation (*n* = 33).

**Figure 2:**
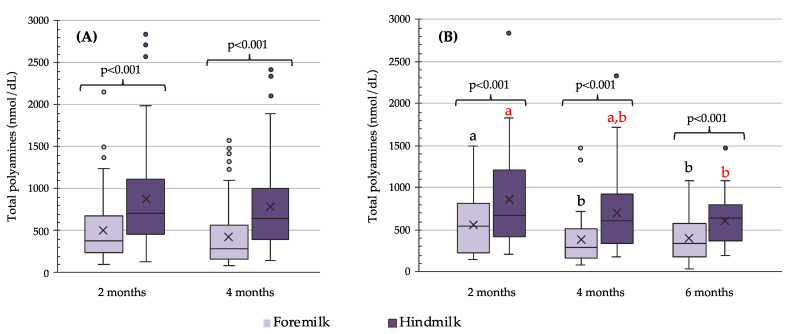
Total polyamine contents (nmol/dL) in foremilk and hindmilk at two and four months of breastfeeding (*n* = 83) (**A**) and during the first six months of breastfeeding (*n* = 33) (**B**). The bottom and top of the box (interquartile range) are the 25th and 75th percentile, respectively. The central line represents the median and X represents the mean. Lines extending vertically from the boxes (whiskers) indicate variability outside the interquartile range. Outliers are plotted as circles. Wilcoxon test was used to compare the polyamine contents between foremilk and hindmilk (**A**,**B**) and along the breastfeeding process (**B**). Different letters in black indicate statistically significant differences among lactation months for foremilk samples and letters in red indicate differences in hindmilk samples, being *p* = 0.009 for foremilk 2 vs. 4 months, *p* = 0.031 for foremilk 2 vs. 6 months, and *p* = 0.011 for hindmilk 2 vs. 6 months.

Polyamines were found in all human milk samples (Figure 2). Total polyamine contents in human milk ranged from 45 nmol/dL to 2841 nmol/dL, although 95% of samples showed levels below 1575 nmol/dL. The variability in total polyamine contents was observed regardless of the month or the phase (foremilk or hindmilk) of breastfeeding, the coefficients of variation always being higher than 50%. Figure 2 shows the total polyamine contents in foremilk and hindmilk at two and four months of breastfeeding (*n* = 83, Figure 2A) and during the first six months of breastfeeding (*n* = 33, Figure 2B). Total polyamine contents were significantly higher in hindmilk than foremilk in all sampling points (*p* < 0.001). Regarding the evolution of polyamines throughout the breastfeeding progress (Figure 2B), a significant decrease was observed in total polyamine levels in foremilk at four and six months with respect to those at two months (*p* = 0.009 and *p* = 0.031, respectively), and in hindmilk between two and six months of lactation (*p* = 0.011).

**Figure 3:**
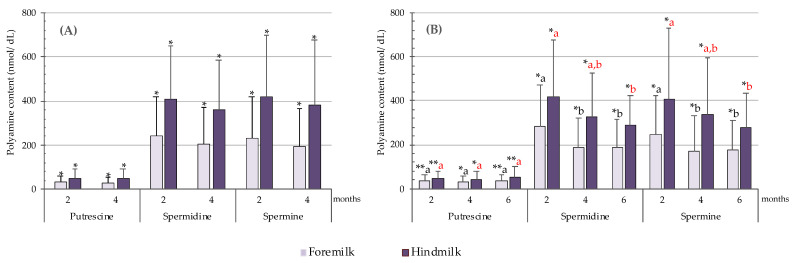
Putrescine, spermidine, and spermine contents (nmol/dL) in foremilk and hindmilk at two and four months of breastfeeding (*n* = 83) (**A**) and during the first six months of breastfeeding (*n* = 33) (**B**). Wilcoxon test was used to compare the polyamine contents between foremilk and hindmilk (**A**,**B**) and along the breastfeeding process (**B**). Asterisks indicate differences between foremilk and hindmilk (* *p* < 0.001 and ** *p* < 0.01). Different letters in black indicate statistically significant differences between lactation months for foremilk samples and letters in red indicate differences in hindmilk samples. For spermidine, *p* = 0.010 for foremilk 2 vs. 4 months, *p* = 0.010 for foremilk 2 vs. 6 months, and *p* = 0.006 for hindmilk 2 vs. 6 months. For spermine, *p* = 0.009 for foremilk 2 vs. 4 months, *p* = 0.049 for foremilk 2 vs. 6 months, and *p* = 0.015 for hindmilk 2 vs. 6 months.

Figure 3 shows the mean contents of putrescine, spermidine, and spermine in foremilk and hindmilk at two and four months of breastfeeding (*n* = 83, Figure 3A) and during the first six months of breastfeeding (*n* = 33, Figure 3B). Putrescine was always the minor polyamine, with mean contents close to 8% of the total polyamines. Mean concentrations of spermidine and spermine were very similar, with ratios of 1.1 regardless of the phase or month of lactation. As can be seen in Figure 3A,B, the levels of the three polyamines were always higher in hindmilk than foremilk, the increases ranging from 1.3- to 2.0-fold depending on the polyamine and the sampling time. According to the Wilcoxon test, these differences between foremilk and hindmilk were statistically significant (*p* < 0.01).

During the first six months of breastfeeding, the mean putrescine concentrations remained constant, whereas those of spermidine and spermine tended to decrease (Figure 3B). Specifically, the Wilcoxon test revealed a statistically significant decrease between months two and four in both spermidine and spermine levels, as well as between two and six months in spermidine and spermine levels (*p* < 0.05).

**Figure 4:**
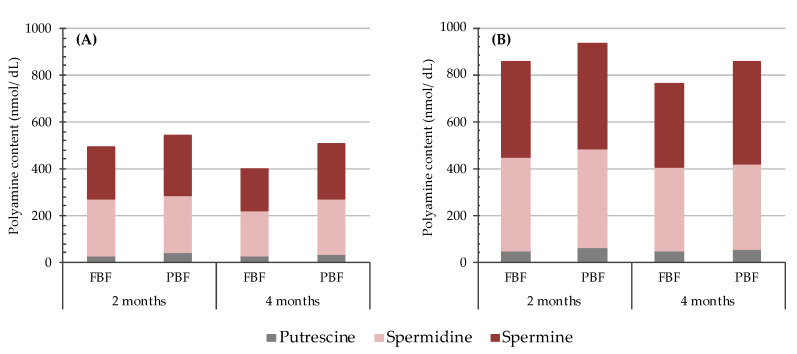
Polyamine levels (nmol/dL) in foremilk (**A**) and hindmilk (**B**) according to the type of breastfeeding (FBF: full breastfeeding and PBF: partial breastfeeding) at two and four months of lactation. FBF group *n* = 60 and PBF group *n* = 23.

Figure 4 shows the contents of the three polyamines according to the type of breastfeeding (full or partial). From a qualitative point of view, the polyamine distribution profile in breast milk did not differ between mothers providing full or partial breastfeeding. A general trend towards higher polyamine levels was associated with partial breastfeeding, although statistically significant differences were only found for spermine at month four. Foremilk and hindmilk samples showed the same trend, although higher contents of the three polyamines were always found in hindmilk, in agreement with the results obtained from the total pool of samples. 

With regard to the characteristics of the mother–child dyad, according to the multiple linear regression model, no significant associations were found between the content of polyamines in human milk with the age and BMI of the mother and with the weight at birth and sex of the infant. Regarding the type of birth, a trend of lower concentrations of polyamines was observed in the milk samples of mothers who gave birth by cesarean section instead of a natural delivery at two months postpartum. However, due to the low number of mothers who delivered by caesarean section, it was not possible to perform a statistical analysis.

## 4. Discussion

It is known that the nutritional composition of breast milk varies considerably and is constantly modified over the course of lactation, mainly in response to the changing requirements of the infant [30,31,32,33]. Although the human milk samples in the current study yielded highly variable polyamine contents, the mean data match those of other reports in the literature (ranging from 150 to 819 nmol/dL) [21,22,23,24]. The wide range of polyamine levels found in human milk in different studies may be attributed to cohort heterogeneity [3,6,21,22,23,24], or to the inter-individuality of each mother–child dyad. Thus, factors such as genetics, ethnic origin, nutritional status, and dietary intake of the mother have been postulated to have an impact on the polyamine content of breast milk [5,6,23,24]. For example, Gómez-Gallego et al. [6] suggested that the geographical region (linked to differences in genetics and dietary patterns) plays a key role, after finding a higher polyamine content in breast milk from mothers in Spain and Finland compared to China and South Africa [27]. However, information about how polyamine intake by the mother can affect breast milk content remains scarce. The study performed by Atiya-Ali et al. [5] showed a positive association between the dietary intake of putrescine, spermidine, and spermine, mainly coming from fruits, with the polyamine content of breast milk. It would be interesting to carry out further studies to better establish the connection between dietary polyamines and their content in human milk.

In accordance with previous reports that describe a progressive decrease in polyamine concentration in human milk during lactation [21,22,23,24], in this study we observed a decline during the first six months. Thus, spermidine decreased significantly by 34% and spermine by 27% between the second and sixth month of breastfeeding. In contrast, putrescine remained practically unchanged during this period. The high growth and differentiation rate of cells during the first months of life may account for the larger amounts of polyamines in milk during the early months of breastfeeding [23]. On the other hand, an increase in the catalytic activity of polyamine oxidase in human milk over the course of lactation could help explain the reduction in polyamine levels after the second month [34].

Regarding the distribution profile of the three polyamines, the results show the predominance of spermidine and spermine, both detected in similar proportions, while the levels of putrescine were always lower, regardless of the sample type. All previously published studies in this field similarly report that putrescine is the least abundant polyamine in human milk [3,6,21,22,23,24], although discrepancies arise about which is the major polyamine, spermidine [5,21,23,24] or spermine [3,6,22]. In any case, a high content of spermine and spermidine in breast milk in the first postnatal months has been linked with a lower risk of developing food allergies in infancy and childhood [10,13,16]. These health-related effects are due to the contribution of spermine and spermidine to the postnatal maturation of the small intestine and the immune system [3,10,11,12,13].

The amount of polyamines in breast milk may vary not only according to the breastfeeding stage, but also during a single feed [5,23]. To our knowledge, the current study is the first to evaluate potential differences in the polyamine concentration between foremilk and hindmilk over the course of lactation. The results of this work showed that polyamine levels were significantly higher (up to two-fold) in hindmilk, especially those of spermidine and spermine, regardless of the breastfeeding month. Regarding the general composition of human milk, it is well established that hindmilk has a higher energy density and greater concentrations of fat and vitamins A and E [30,32,35,36]. Moreover, the fact that hindmilk remains in the mammary gland for longer in the presence of active endo- and exopeptidases (i.e., enzymes responsible for breaking down the amino acids of milk peptides) could lead to a higher content of free amino acids [35,36]. Specifically, Sadelhoff et al. [36] reported that hindmilk contains more arginine, which is the precursor amino acid of ornithine, a key substrate for the endogenous synthesis of polyamines [37,38]. The higher content of polyamines in hindmilk reinforces the importance of full feeds for infants if they are to benefit from all the nutrients provided by breast milk.

According to the World Health Organization, exclusive breastfeeding is recommended for infants during the first six months of life, followed by breast milk combined with foods for up to two years [39]. However, there is a growing tendency towards the partial breastfeeding of newborns [40]. The type of breastfeeding could also affect the composition of human milk, although it has been scarcely investigated so far. Jia et al. [41] reported that the lactose and protein content were significantly higher in the milk of mothers that partially breastfed, compared with those providing full breastfeeding during the first four months postpartum. In the current study, slightly higher levels of polyamines were observed in the milk of mothers providing partial rather than full breastfeeding, although significant differences were only found for one polyamine at four months. As the participants were always encouraged to practice full breastfeeding, only a few mothers partially breastfed (*n* = 23), which may limit the drawing of any solid conclusions.

According to Gómez-Gallego et al. [6], the type of birth, whether natural or by cesarean section, seems to affect the concentration of polyamines in human milk. These authors reported that mothers who delivered their baby by cesarean section produced milk with a lower polyamine content [6]. In our study, although only 5% of the mothers underwent caesarean section, a trend towards lower levels of polyamines in milk was also observed. However, due to the low number of samples, it is not possible to draw conclusions about the influence of the type of delivery on the polyamine levels of human milk. Further studies are needed, not only to confirm these outcomes, but also to analyze why polyamine levels may vary according to the birth typology. Finally, no significant associations were found between other factors related to the mother–child dyad (age and BMI of the mother, birth weight, and sex of the infant) and the polyamine levels in human milk. Although the available information regarding the influence of these factors in the levels of polyamines in human milk is still scarce, Atiya-Ali et al. [24] reported lower contents of polyamines in the milk of mothers with obesity compared to those with normal weight.

## 5. Conclusions

The polyamine content of breast milk from Mexican mothers varied considerably, and generally decreased during the course of lactation. This study reports for the first time that the amount of polyamines in human milk can change during a single feed, with the levels being significantly higher in hindmilk than in foremilk, especially those of spermidine and spermine. A general trend towards higher levels of polyamines in milk was observed in mothers who provided partial rather than full breastfeeding, although only spermine was statistically higher. Overall, these results indicate that polyamines in human milk may change, both during a single feed (foremilk versus hindmilk) and throughout lactation. The various health-related effects attributed to polyamines call for further studies to properly elucidate their role in infant development. More information is also needed on how polyamines in human milk are affected by different factors related to lactation, the mother, and the infant. In particular, the influence of the mother’s polyamine intake deserves more detailed study, as a polyamine-enriched diet could be an effective strategy to increase the polyamine content of breast milk.

## Figures and Tables

**Table 1:** Sociodemographic characteristics of the mother–child dyad.

Mother	Total Cohort (*n* = 83)	Subgroup (*n* = 33)
Age (years)	23.5 ± 4.5	23.0 ± 4.6
BMI (mg/kg^2^) *	25.0 ± 5.7	24.9 ± 6.5
Education level		
Incomplete junior high school or lower	18 (22%)	6 (18%)
Complete junior high school	30 (36%)	12 (36%)
Complete high school or higher	35 (42%)	15 (46%)
Occupation		
Housewife	69 (83%)	31 (94%)
Employee/merchant	8 (10%)	1 (3%)
Student	3 (3.5%)	1 (3%)
Unemployed	3 (3.5%)	-
Marital status		
Married	20 (24%)	8 (24%)
Free union	50 (60%)	20 (61%)
Single/separated	13 (16%)	5 (15%)
**Infant**		
Birth weight	3210.3 ± 340.1	3195.9 ± 333.4
Sex		
Female	29 (35%)	12 (36%)
Male	54 (65%)	21 (64%)
Delivery type		
Natural birth	79 (95%)	31 (94%)
Cesarean section	4 (5%)	2 (6%)

Data are presented as mean ± SD and as frequencies and percentages. * BMI: body mass index at two months postpartum.
